# Polarization‐Multiplexed Double‐Helix/Airy Metalens for Depth‐Resolved Texture Imaging With Extended Depth‐of‐Field

**DOI:** 10.1002/nap2.70311

**Published:** 2026-10-06

**Authors:** Xinyi Tian, Zhanhong Zhou, Bohan Xiao, Li Yao, Zhihong Liu, Tianyun Wang, Yuhong Luo, Jun Wang, Yujie Xia, Chao Shen, Junwen Zhang, Nan Chi, Ziwei Li

**Affiliations:** ^1^ College of Future Information Technology Fudan University Shanghai China; ^2^ Key Laboratory for Information Science of Electromagnetic Waves (MoE) Fudan University Shanghai China

**Keywords:** computational imaging, depth imaging, extended depth‐of‐field, polarization‐multiplexed metalens

## Abstract

Simultaneously acquiring high‐fidelity texture and reliable depth cues over an extended working range remains a central challenge for compact passive imaging systems. Conventional point‐spread‐function (PSF)‐engineered optics usually rely on a single optical response to perform both axial encoding and image formation, creating an inherent trade‐off between depth discriminability and lateral image quality. Here, we demonstrate a polarization‐multiplexed multifunctional metalens that decouples depth encoding from texture observation within a single titanium dioxide planar device. By jointly designing propagation and Pancharatnam–Berry geometric phases, the two circular polarization channels are assigned to double‐helix (DH) PSF response for depth encoding and Airy PSF for extended‐depth‐of‐field (EDOF) texture observation, respectively. The Airy‐channel target scale serves as an internal reference for depth‐feature extraction. The DH‐derived depth further guides Airy‐PSF selection and enables non‐blind texture deconvolution. Experiments on multi‐depth letter targets and textured objects demonstrate depth‐resolved texture recovery over a 20–70 cm working distance, with improved contrast, sharpness, and structural similarity. This function‐decoupled DH/Airy architecture uses complementary PSF channels to mitigate the trade‐off between depth discriminability and lateral image quality, providing a compact optical‐computational strategy for depth‐aware EDOF imaging in miniaturized metalens systems.

## Graphical abstract

To address the trade‐off between depth discriminability and texture preservation in PSF‐engineered imaging, a polarization‐multiplexed metalens is developed to decouple depth estimation from extended‐depth‐of‐field texture acquisition. The DH‐derived depth guides depth‐matched Airy PSF selection for non‐blind texture recovery, while computational fusion enables depth‐resolved, texture‐preserving representations of multi‐depth scenes and complex objects.

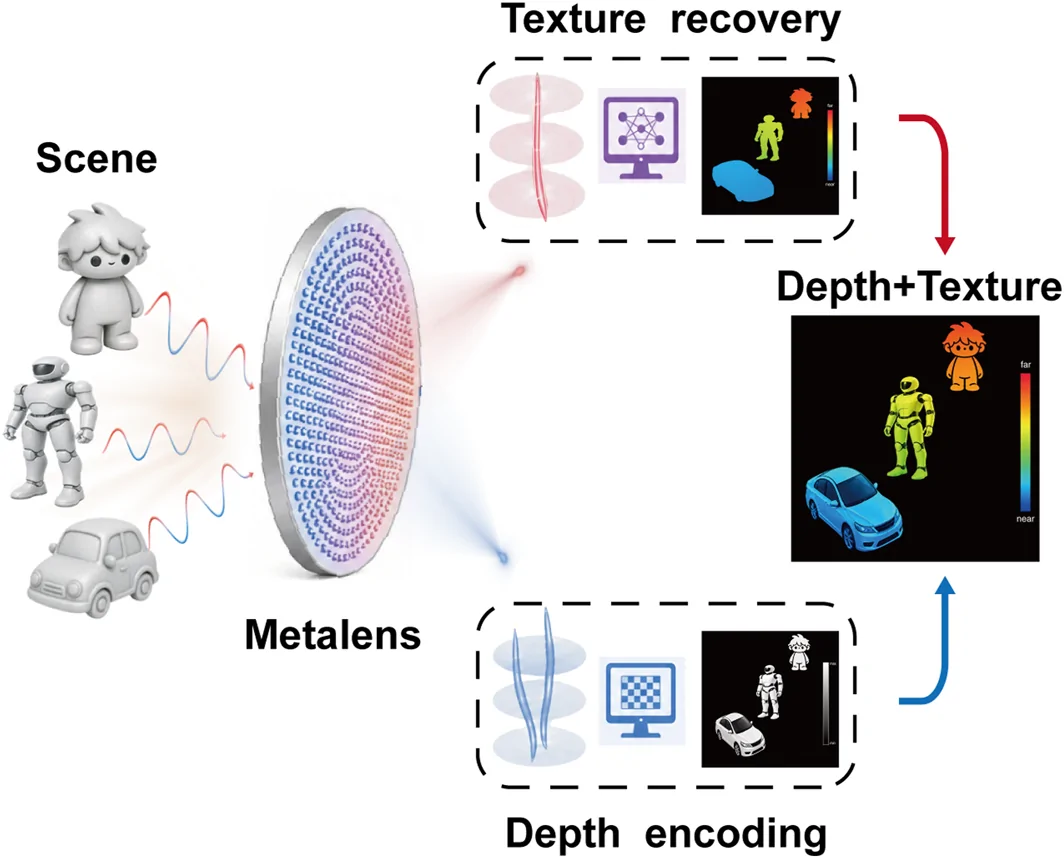

## 1 Introduction

Three‐dimensional imaging plays an essential role in machine vision, intelligent sensing, and human–machine interaction [1, 2, 3, 4]. In practical compact‐vision scenarios, an imaging system is expected to provide not only geometric depth, but also object edges, textures and appearance details that support recognition, segmentation, and interpretation. Point‐spread‐function (PSF) engineering provides an attractive single‐aperture route to passive depth sensing [5] by encoding axial position into image features such as defocus, lateral displacement, rotation [6, 7], or multi‐lobe spacing [8, 9]. However, the central difficulty is not depth encoding alone, but preserving lateral texture fidelity while maintaining depth discriminability over a large axial range. This depth–texture conflict is especially important for miniaturized optical systems, where the optical front end must provide both geometric cues and usable appearance information within a limited form factor.

Metalenses, composed of subwavelength nanostructures, provide an ultrathin platform for implementing complex phase, polarization [10, 11, 12], and wavefront responses [13, 14], and have become powerful optical elements for PSF‐engineered computational imaging [15, 16, 17, 18, 19]. Among engineered PSFs, the double‐helix (DH) PSF has been widely used in passive ranging and metalens‐based depth imaging [20, 21, 22, 23, 24, 25], because its two‐lobe response rotates, shifts, or changes spacing with propagation distance [6, 7, 21, 26]. This depth‐dependent twin‐lobe structure provides strong axial cues for calibration‐based depth estimation. However, the same mechanism also introduces a fundamental limitation. Since the DH response is spatially extended, object information is distributed into two depth‐dependent replicas, which can blur edges, mix texture features, and reduce lateral‐detail fidelity for complex targets. Thus, DH‐type PSFs obtain depth sensitivity by sacrificing spatial compactness, creating an intrinsic trade‐off between axial encoding and texture preservation.

Polarization multiplexing provides an additional degree of freedom to address this single‐PSF limitation [27]. By jointly designing propagation phase and Pancharatnam–Berry geometric phase, two orthogonal circular polarization channels can be assigned independent wavefront responses in one metasurface [10, 28, 29, 30], enabling compact multifunctional optical encoding [31, 32]. Recent advances in achromatic and broadband metalenses [33, 34], on‐chip metasurface integration [35, 36, 37, 38], monocular or binocular metasurface cameras [39, 40, 41], diffraction–polarization depth fusion [42], adaptive rotational depth mapping [43], and polarization computational imaging have demonstrated the broad potential of metasurface optics for compact perception [28, 44, 45, 46]. Nevertheless, existing metalens‐based or polarization‐multiplexed 3D imaging schemes usually allocate the available channels mainly to depth encoding, disparity extraction, polarization contrast, or multi‐degree‐of‐freedom sensing [39, 40, 42, 43]. Texture is often treated as a by‐product of the depth‐imaging process rather than optimized as an independent optical task. For extended depth‐encoding responses such as DH PSFs, conventional deconvolution or learning‐based reconstruction may partially restore texture, but these approaches do not remove the optical conflict between depth encoding and texture preservation [47, 48, 49]. A more efficient strategy is to physically separate these two functions into two complementary PSFs, where one depth‐sensitive PSF provides axial encoding while another EDOF PSF preserves texture.

Here, we propose and experimentally demonstrate a polarization‐multiplexed DH/Airy metalens that realizes this complementary‐PSF strategy in a single TiO_2_ planar device. Through the joint design of propagation and Pancharatnam–Berry geometric phases, the two circular polarization channels are assigned to DH depth encoding and Airy EDOF texture observation, respectively [50]. The DH channel provides depth‐dependent features for axial estimation, whereas the Airy channel offers a laterally more compact and depth‐tolerant response for texture acquisition. The DH‐derived depth further guides Airy‐PSF selection and enables non‐blind texture deconvolution, converting depth estimation into a physical prior for depth‐aware texture recovery. We experimentally validated depth‐resolved texture recovery using multi‐depth letter targets and textured objects over a 20–70 cm working distance, showing improved contrast and sharpness after depth‐guided restoration. This function‐decoupled DH/Airy architecture provides a compact optical‐computational route for depth‐aware EDOF imaging and offers a complementary‐PSF design strategy for mitigating the trade‐off between depth encoding and texture preservation in miniaturized metalens systems.

## 2 Results

### 2.1 Principle of Depth‐Resolved Imaging With a Complementary‐PSF Metalens

In conventional PSF‐engineered passive depth imaging, a single optical response is usually required to encode axial information and preserve lateral object details at the same time, yet these two requirements are intrinsically conflicting. A depth‐sensitive PSF should vary strongly with axial position so that depth can be reliably inferred from the recorded image. However, such a response is often spatially extended or highly structured, which spreads object information over a large detector area and degrades edge sharpness, texture fidelity, and object recognizability. For example, using a DH PSF alone for both depth encoding and image formation inevitably introduces a trade‐off between depth discriminability and lateral texture preservation.

To mitigate this trade‐off, we propose a complementary‐PSF strategy instead of forcing a single PSF to perform both tasks. Figure 1 illustrates the principle of the polarization‐multiplexed DH/Airy metalens for depth‐resolved imaging with EDOF. As shown in Figure 1a, the incident field is decomposed into two circular polarization components, and the two spin channels are assigned different optical functions within one planar metalens. The left‐handed circular polarization (LCP) channel is designed to generate a DH response for depth encoding, whereas the right‐handed circular polarization (RCP) channel is designed to generate an Airy response for extended‐depth‐of‐field texture observation. The two PSFs are complementary in their physical roles: The DH channel converts axial displacement into a measurable geometric feature, such as the separation or relative position of the two encoded replicas. The Airy channel, by contrast, maintains a laterally more compact and depth‐tolerant main‐lobe response, so that object boundaries and texture information can be observed over an extended working range. In this way, axial encoding and texture acquisition are physically separated at the optical‐architecture level.

**FIGURE 1:**
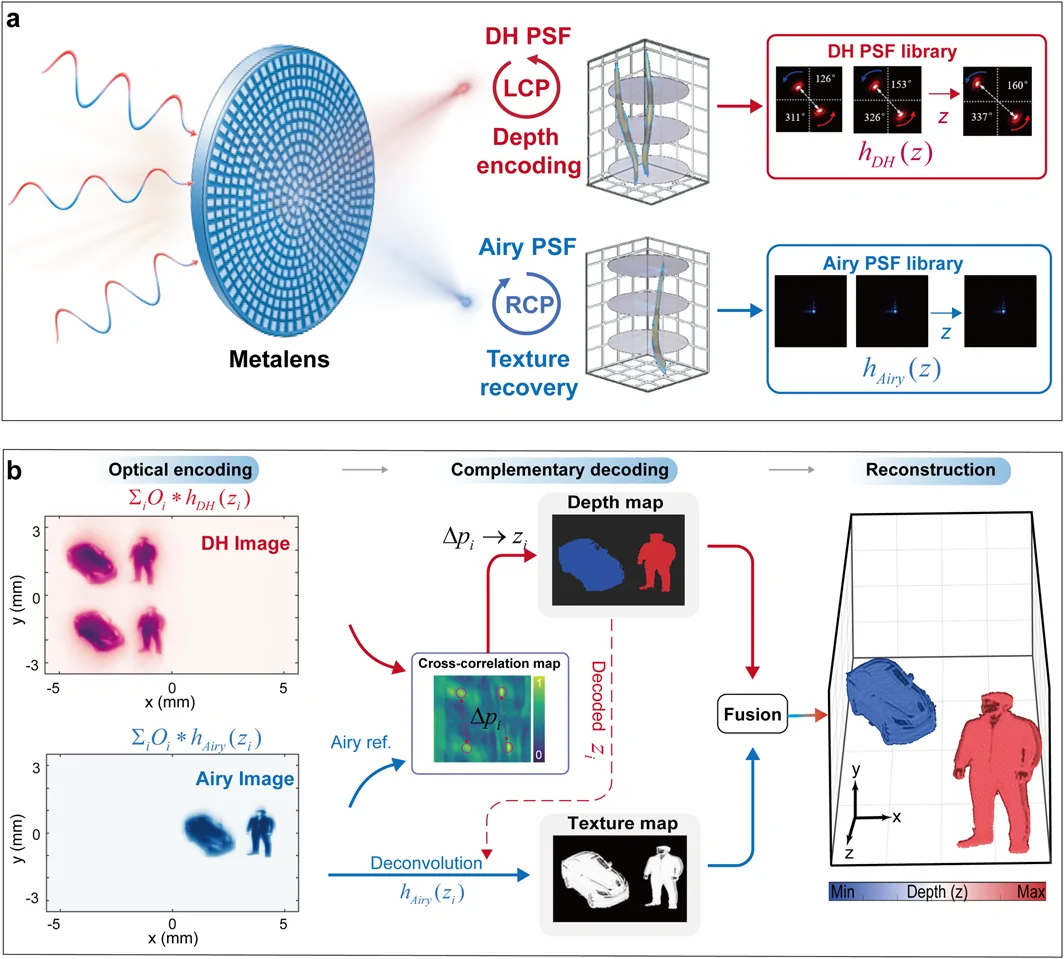
Concept of the polarization‐multiplexed DH/Airy metalens for depth‐guided texture recovery. (a) Dual‐channel optical encoding, where the LCP channel forms a DH image for depth encoding and the RCP channel forms an Airy EDOF image for texture acquisition. (b) Simulation‐based demonstration of the complementary decoding workflow, in which Airy‐assisted DH depth extraction guides depth‐conditioned Airy‐channel texture deconvolution and depth–texture point‐cloud reconstruction.

The complementary relationship between the two channels was first evaluated numerically using simulated DH and Airy images generated with their depth‐dependent PSFs, as shown in Figure 1b. The Airy image is first used as an internal structural reference for locating the DH‐encoded replicas. This step is particularly useful when the two DH replicas overlap strongly or when the object has an irregular silhouette, because the Airy observation provides a single‐image estimate of the object support and target scale. By correlating the Airy‐derived template with the DH image, the centers of the two DH replicas can be localized more robustly, and their feature spacing can then be converted into an axial position through the calibrated DH feature–depth relation. Thus, the Airy channel does not merely provide an independent texture image; it also assists the extraction of depth features from the DH channel. After depth estimation, the information flow is reversed: the DH‐derived depth becomes a physical prior for texture recovery in the Airy channel. Since the Airy PSF still changes with axial position, directly restoring the Airy image without depth information would require blind or poorly constrained deconvolution. In our framework, the depth estimated from the DH channel selects the corresponding measured Airy PSF from the PSF library. The Airy texture recovery is therefore reformulated as a depth‐conditioned non‐blind deconvolution problem with a known physical kernel. This depth‐guided restoration improves the contrast and sharpness of the Airy observation while avoiding the texture duplication caused by the DH response. Finally, the recovered Airy texture and the DH‐estimated depth are fused to generate a depth‐resolved texture representation. For multi‐depth or layer‐wise targets, the method assigns recovered textures to their corresponding axial positions, producing a depth–texture point‐cloud representation that preserves both geometric depth cues and lateral appearance details.

After defining the complementary roles of the two channels, we next design a spin‐decoupled metalens that can carry the DH and Airy responses simultaneously. Figure 2 presents the detailed phase‐synthesis strategy for the two spin channels. Specifically, the LCP channel is assigned to the DH response for depth encoding, whereas the RCP channel is assigned to the Airy response for texture observation.

**FIGURE 2:**
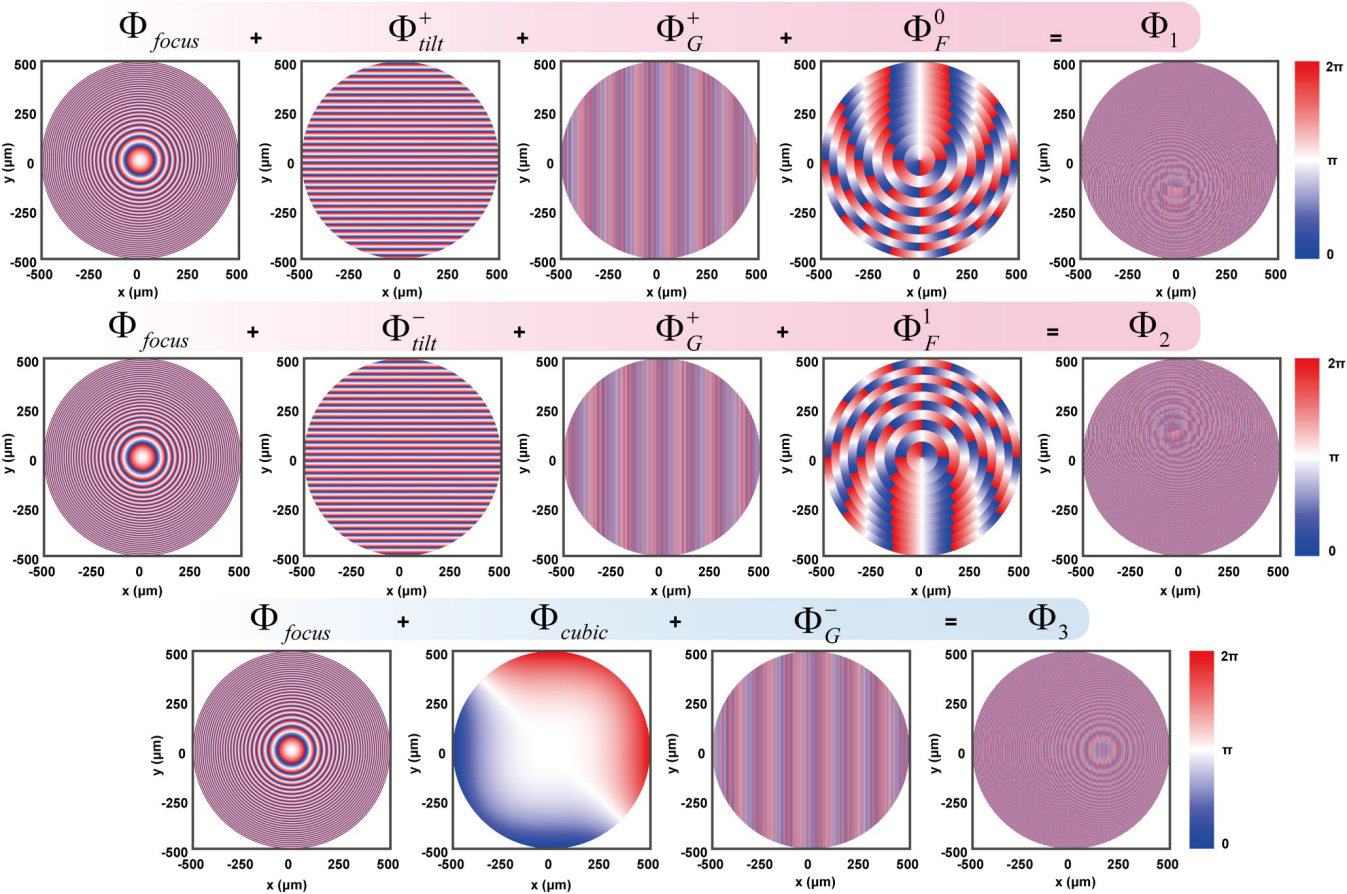
Detailed phase‐synthesis procedure for the spin‐decoupled DH/Airy metalens. The first and second rows show the construction of Φ_1_ and Φ_2_ from the focusing phase, opposite steering phases, a common positive linear‐gradient phase, and complementary Fresnel‐zone azimuthal phases. The third row shows the construction of Φ_3_ from the focusing, cubic, and negative linear‐gradient phases.

The corresponding LCP and RCP target phases are obtained as follows:

(1) $$\Phi_{\text{LCP}}\left(x,y\right)=\arg\left[\exp\left(i\Phi_{1}\right)+\exp\left(i\Phi_{2}\right)\right],$$

(2) $$\Phi_{\text{RCP}}\left(x,y\right)=\arg\left[\exp\left(i\Phi_{3}\right)\right],$$

here the phase profiles $\Phi_{1}$ and $\Phi_{2}$ contain a common focusing phase $\Phi_{\text{focus}}$ and the same positive linear‐gradient phase $\Phi_{G}^{+}$, while incorporating opposite steering terms $\Phi_{\text{tilt}}^{+}$ and $\Phi_{\text{tilt}}^{-}$, and Fresnel‐zone azimuthal phases $\Phi_{F}^{0}$ and $\Phi_{F}^{1}$, respectively. Their equal‐amplitude coherent superposition defines the target phase of the LCP channel. $\Phi_{3}$ is constructed by combining a focusing phase, a cubic phase $\Phi_{\text{cubic}}$, and a negative linear‐gradient phase $\Phi_{G}^{-}$. The cubic phase improves defocus tolerance because the quadratic phase introduced by defocus can be transformed into a lateral shift of the cubic‐phase response, thereby reducing defocus‐induced PSF broadening [51]. Owing to the finite aperture of the metalens, the resulting field forms a truncated, finite‐energy Airy‐like PSF [52], whose profile remains comparatively stable over the designed axial range. Explicit analytical expressions for these constituent phase terms are provided in Supporting Information S1: Note 2.

For an anisotropic meta‐atom, the two circular polarization target phases are related to the propagation phases $\phi_{x}$, $\phi_{y}$ along the two orthogonal eigenaxes and the in‐plane rotation angle θ by

(3) $$\phi_{x}=\frac{\Phi_{\text{LCP}}+\Phi_{\text{RCP}}}{2},$$

(4) $$\phi_{y}=\phi_{x}-\pi ,$$

(5) $$\theta =\frac{\Phi_{\text{LCP}}-\Phi_{\text{RCP}}}{4}.$$

The resulting spatial distributions of $\phi_{x}$, $\phi_{y}$, and θ are shown in Figure 3a–c. A detailed Jones matrix derivation of the spin‐multiplexed phase relation is provided in Supporting Information S1: Note 2. To implement these phase profiles, anisotropic TiO_2_ nanobricks on a SiO_2_ substrate are used as the meta‐atoms, as illustrated in Figure 3d. The nanobrick geometry is defined by the period *P*, height *H*, length *L*, width *W*, and in‐plane rotation angle *θ*. The anisotropic TiO_2_ nanobricks are then selected from parameter sweeps to provide the required propagation phases while maintaining high polarization conversion efficiency (PCE), as shown in Figure 3e,f. The material dispersion and complete phase and transmission maps used for meta‐atom selection are provided in Supporting Information S1: Note 1 and Figure S1.

**FIGURE 3:**
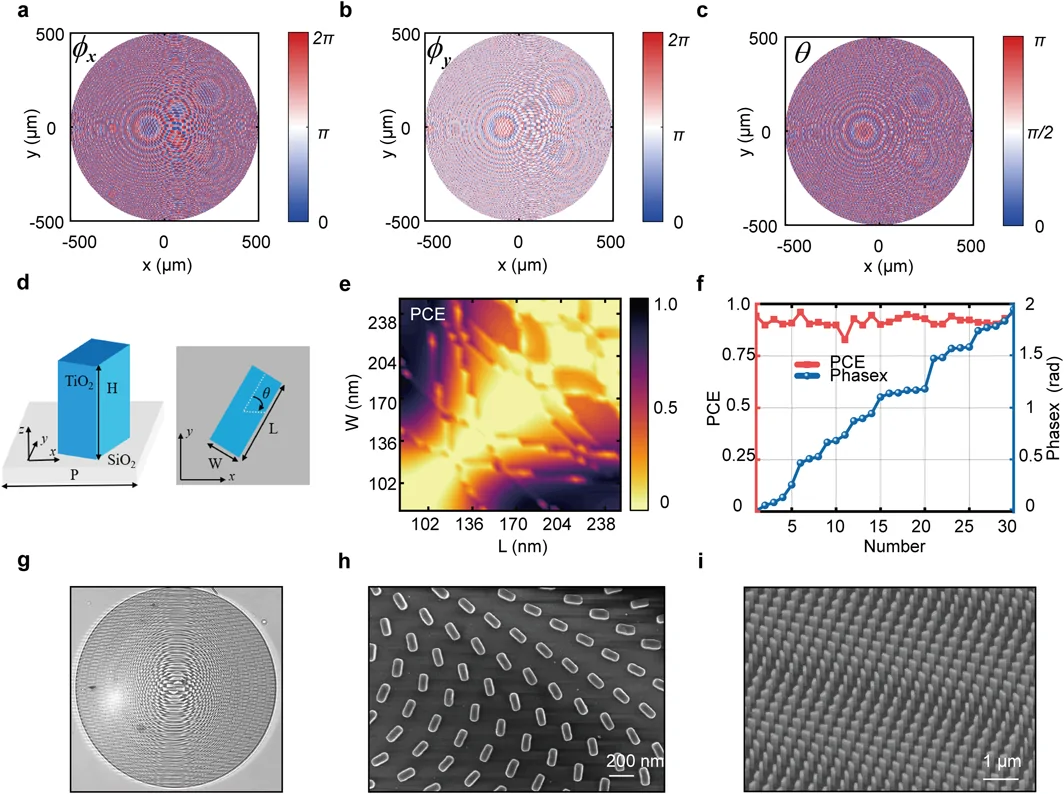
Design and fabrication of the spin‐decoupled DH/Airy metalens. (a–c) Calculated eigenaxis phase distributions and in‐plane rotation‐angle map. (d) Schematic of the anisotropic TiO_2_ nanobrick meta‐atom on a SiO_2_ substrate. (e) Simulated polarization conversion efficiency as a function of nanobrick length and width. (f) Phase response and polarization conversion efficiency of the selected meta‐atoms. (g) Optical microscope image of the fabricated metalens. (h, i) Local SEM images showing the nanobrick arrangement and orientation distribution.

Figure 3g shows an optical microscope image of the fabricated metalens. The fabricated device and scanning electron microscopy (SEM) images in Figure 3h,i further confirm the designed aperture pattern, orientation distribution, and large‐area fabrication uniformity, establishing the physical basis for implementing the dual‐channel DH/Airy PSFs.

### 2.2 Experimental Characterization of Complementary DH/Airy PSF Channels

After implementing the spin‐decoupled metalens, we experimentally characterized the two PSF channels to verify their complementary functions. Figure 4 compares the measured DH and Airy responses during axial scanning. For the DH channel, the *xz* and *yz* projections in Figure 4a both show continuously evolving two‐branch trajectories during axial propagation, indicating that this channel encodes depth information into the spatial distribution of two spots. The *xy* sections at different *z* planes show clear twin‐spot structures, and the twin‐spot spacing, connecting‐line angle and left/right spot positions vary with depth. Figure 4c shows image‐plane transverse intensity profiles along the line connecting the two DH peaks. The peak‐to‐peak spacings between the twin‐spot profiles at *z*1, *z*2, and *z*3 are 2.66, 2.87, and 3.31 mm, respectively, reflecting the spatially extended response of the DH PSF on the detector plane. Furthermore, the centers of the two DH lobes were determined by subpixel intensity‐weighted centroid localization and their axial trajectories were fitted independently with circles. The fitted trajectory radii are 204.67 and 205.71 μm on the sensor plane, respectively. The two lobes cover fitted rotation ranges of 313.68° and 308.49°, where the decimal values reflect subpixel localization and fitting rather than 0.01° absolute angular accuracy.

**FIGURE 4:**
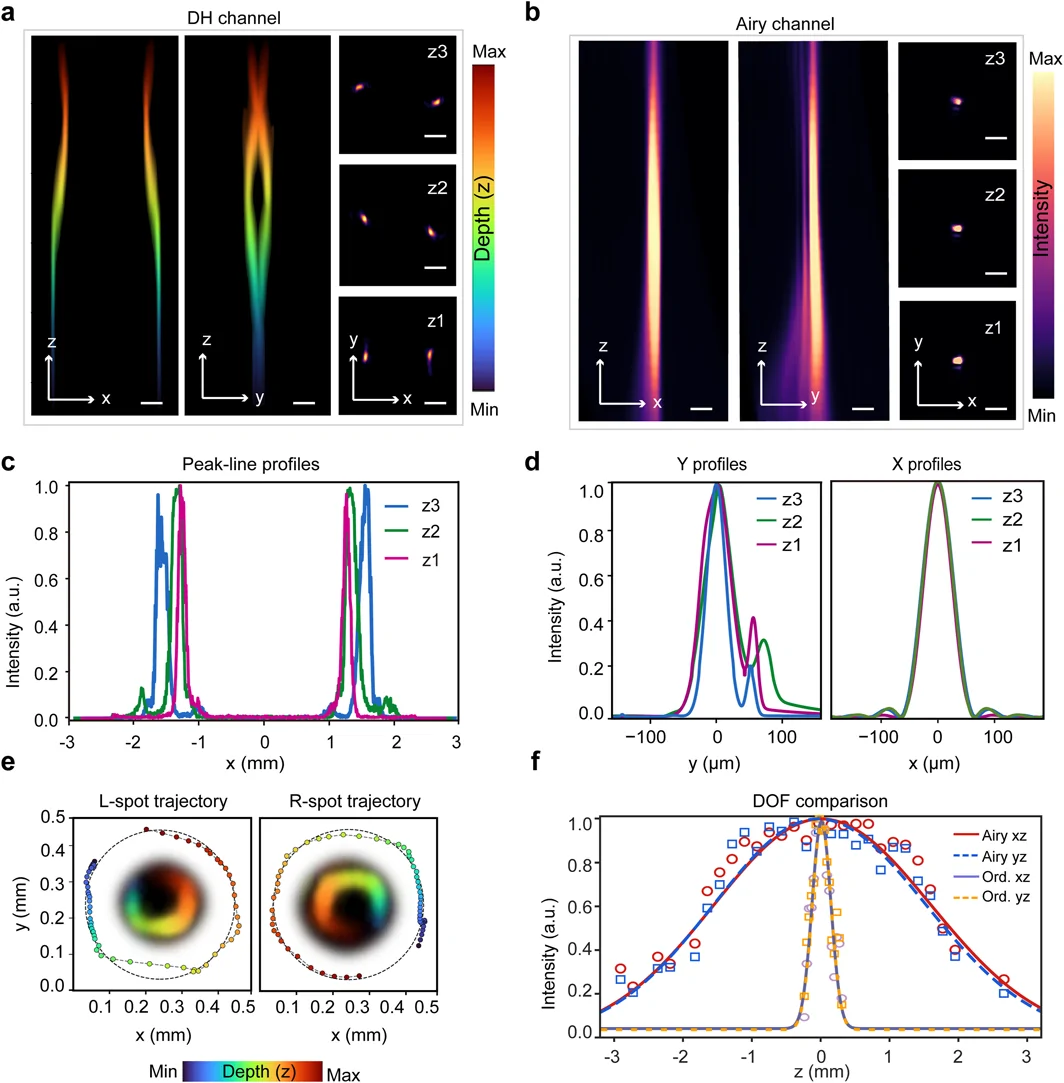
Experimental characterization of the complementary DH and Airy PSF channels. (a, b) Measured *xz*/*yz* projections and representative *xy* sections of the DH and Airy PSFs at different axial positions. (c) Peak‐line profiles of the DH PSF. (d) Transverse intensity profiles of the Airy main lobe. (e) Measured trajectories and circular fits of the two DH lobes during axial scanning. (f) Measured DOF comparison between the Airy channel and an ordinary focused lens, showing an approximately 11‐fold extension of the depth of field.

In contrast, the Airy channel provides a more compact and depth‐tolerant response. The *xz* and *yz* projections in Figure 4b show a continuous single‐branch axial response, and the *xy* sections at *z*1, *z*2, and *z*3 retain a dominant main lobe with weak sidelobes. The transverse profiles in Figure 4d show that the *y*‐direction main‐lobe full width at half maximum (FWHM) remains approximately 57–62 μm, whereas the *x*‐direction FWHM remains approximately 34.4–49.7 μm over the three representative depths. To quantify how effectively each polarization channel concentrates its optical power into the intended PSF region, we evaluated the target‐region power‐concentration efficiency. The simulated efficiencies are 75.11% for the DH channel and 99.57% for the Airy channel (Supporting Information S1: Note 5).

Figure 4f further compares the measured axial response of the Airy channel and a conventional focusing lens under the same focal length, effective aperture, axial scanning, and detection conditions. Based on the FWHM of the axial intensity response, the Airy channel exhibits an approximately 11‐fold larger effective depth of field while maintaining a compact main‐lobe response. Numerical PSF and modulation transfer function (MTF) analyses show comparable focal‐plane main‐lobe widths for the two channels, whereas the Airy channel exhibits a moderate reduction in best‐focus spatial‐frequency response in exchange for a slower degradation with defocus (Supporting Information S1: Note 6). This enhanced defocus tolerance enables stable texture acquisition over an extended axial range and provides the depth‐dependent PSFs required for subsequent non‐blind deconvolution.

### 2.3 Depth‐Guided Texture Recovery for Depth‐Resolved EDOF Imaging

After characterization of the DH and Airy channels, we next evaluated whether the depth information encoded by the DH response can be converted into a useful physical prior for Airy‐channel texture recovery. The experiments in this section were designed to progressively validate three key capabilities of the proposed framework: calibrated axial localization from the DH channel, depth‐conditioned non‐blind restoration in the Airy channel, and fusion of the recovered depth and texture into depth‐resolved representations. We first used controlled multi‐depth targets to quantify the feature–depth relationship and verify the reconstruction workflow. We then extended the method to textured objects with more complex silhouettes and appearance details. Finally, we examined irregular three‐dimensional objects to assess whether the complementary‐PSF strategy can preserve object contours and local texture features beyond simple planar patterns.

#### 2.3.1 Depth Calibration and Airy‐Assisted Reconstruction of Multi‐Depth Targets

Figure 5 demonstrates the calibration and reconstruction workflow using measured targets placed at different axial positions. The dual‐channel optical configuration is shown in Figure 5a. Under circular polarization analysis, the same object is recorded through two complementary responses: the DH channel produces depth‐dependent twin replicas, whereas the Airy channel provides a single EDOF observation that better preserves the object support. Before reconstructing unknown targets, we first established the axial calibration curve by measuring the DH peak spacing at known object distances over a 20–70 cm working range. As shown in Figure 5b, the measured peak spacing follows the calculated depth‐dependent trend, providing a direct mapping from the DH geometric feature to object depth.

**FIGURE 5:**
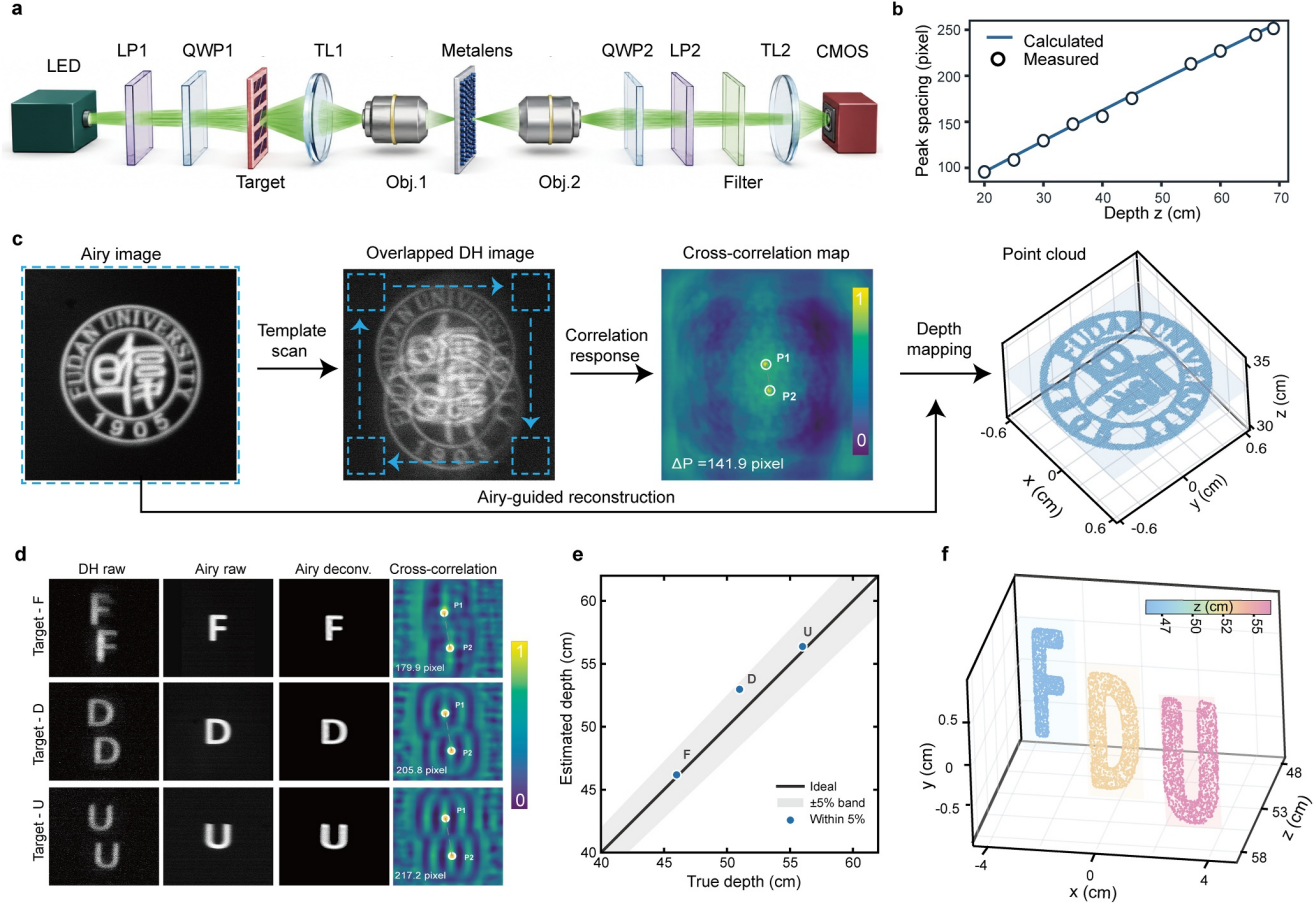
Depth extraction and point‐cloud reconstruction of measured targets. (a) Dual‐channel optical setup. (b) DH peak‐spacing calibration over a 20–70 cm working distance. (c) Airy‐assisted depth extraction from an overlapped emblem target. (d) DH images, Airy images, depth‐selected deconvolution results, and cross‐correlation maps for F, D, and U targets. (e) Estimated depth versus true depth. (f) Point‐cloud reconstruction results of the F, D, and U letters.

The Airy image is then used to assist DH feature extraction. For the overlapped emblem target in Figure 5c, the DH image contains two spatially shifted replicas whose centers are not immediately obvious because of the extended DH response and the internal structure of the object. Instead of detecting the DH peaks solely from local intensity maxima, we used the Airy observation as an internal structural template and scanned it across the DH image. The resulting cross‐correlation map identifies two response maxima, *P*1 and *P*2, corresponding to the centers of the DH‐encoded replicas. The two correlation peaks were localized using subpixel intensity‐weighted centroid estimation, yielding continuous peak coordinates. Their two‐dimensional Euclidean separation gives Δ*P* = 141.9 pixels, which is then converted into the target depth using the calibrated peak‐spacing–depth relation. This procedure shows how the Airy channel improves the robustness of DH feature localization, especially when the DH replicas are partially overlapped or contain complex object structures.

We further applied the same imaging workflow to F, D, and U letter targets positioned at different depths. Figure 5d compares the raw DH observations, raw Airy observations, depth‐selected Airy deconvolution results, and correlation maps. The DH channel provides the axial feature spacing, while the Airy channel supplies the texture‐bearing image for restoration. After the depth was estimated from the DH spacing, the corresponding measured Airy PSF at that axial position was selected from the PSF library and used for non‐blind deconvolution of the Airy image. The corresponding relative errors are 0.39%, 3.87%, and 0.67%, respectively, giving a normalized mean absolute error (NMAE) of 1.64%, calculated according to the definitions in Supporting Information S1: Note 7. The ± 5% band in Figure 5e provides a visual reference for the depth estimates. The restored textures were then assigned to their estimated axial positions to form the depth–texture point‐cloud representations in Figure 5f.

#### 2.3.2 Depth‐Guided Texture Recovery and Point‐Cloud Reconstruction of Complex Objects

We next examined whether the same depth‐guided restoration strategy remains effective for objects with irregular silhouettes and richer texture content. Figure 6 shows the results for Doll, Robot, and Car targets. In the DH channel, each object is encoded into a pair of depth‐dependent replicas. Although these replicas provide useful axial cues, their spatially extended and duplicated appearance makes them unsuitable for direct texture interpretation. In contrast, the Airy channel records a single EDOF observation with more compact object support, but its raw image still contains depth‐dependent blur and reduced contrast. The complementary decoding strategy therefore uses the DH image primarily for depth estimation and the Airy image primarily for texture recovery.

**FIGURE 6:**
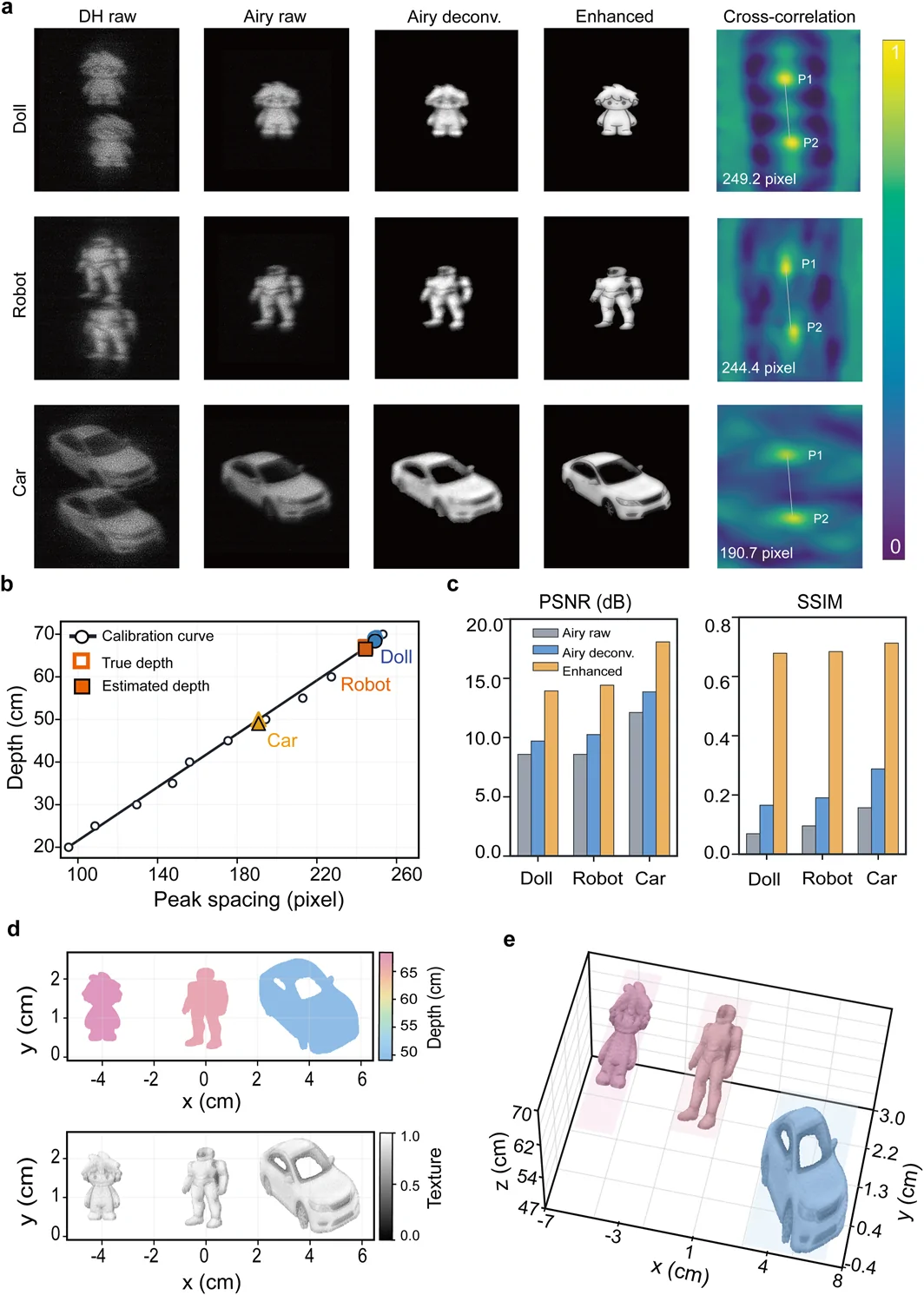
Texture‐preserving reconstruction of complex textured objects. (a) DH raw, Airy raw, Airy deconvolution, enhanced texture, and correlation‐derived peak‐spacing results for Doll, Robot, and Car. (b) Axial depth inference of the three targets using the peak‐spacing‐depth calibration curve. (c) PSNR and SSIM comparisons among Airy raw, Airy deconvolution, and enhanced results. (d) Depth map obtained from the DH channel (top) and texture map recovered from the Airy channel (bottom). (e) Reconstruction after depth–texture fusion.

For each object, the Airy observation was used as a structural template to locate the two DH‐encoded replicas through cross‐correlation. The extracted peak spacings were then mapped to axial positions using the calibration curve, as shown in Figure 6b. The estimated depths agree well with the true object positions, confirming that DH feature extraction remains reliable for non‐letter targets with more complex appearance. The inferred depth was subsequently used to select the corresponding measured Airy PSF, enabling depth‐conditioned non‐blind deconvolution of the Airy raw image. Compared with the raw Airy observations, the deconvolved results exhibit clearer object boundaries, enhanced local contrast, and improved texture visibility.

The quantitative results in Figure 6c further support this improvement. Relative to the Airy raw images, Airy deconvolution improves the object‐region‐of‐interest (ROI) peak signal‐to‐noise ratio (PSNR) of Doll, Robot, and Car by 1.07, 1.59, and 1.65 dB, respectively, and increases structural similarity index (SSIM) by 0.090, 0.089, and 0.123. Ground‐truth (GT)‐guided enhancement further improves texture consistency, with object‐ROI PSNR reaching 13.24, 13.70, and 17.17 dB and SSIM reaching 0.636, 0.641, and 0.668 for the three objects. No‐reference image‐quality metrics show a more pronounced enhancement in perceptual clarity: root‐mean‐square (RMS) contrast increases by 27.9%, 38.5%, and 43.7%, while Tenengrad sharpness increases by 73.2%, 89.6%, and 121.1% for the three objects, respectively. These results indicate that the DH‐derived depth prior provides a useful physical constraint for restoring the Airy‐channel texture. The reference‐guided enhanced results are included as an upper‐bound visualization of appearance refinement, but the primary recovery pathway relies on the measured Airy image and the depth‐selected Airy PSF.

The DH‐estimated depth and Airy‐recovered texture were fused to generate the depth–texture representations shown in Figure 6d,e. The depth map records the axial positions inferred from the DH channel, whereas the texture map is constrained by the Airy‐channel object support and restored appearance. Their fusion produces a three‐dimensional representation in which different targets are assigned to their corresponding depths while retaining recognizable lateral appearance.

#### 2.3.3 Depth–Texture Rendering of 3D Objects

We further tested the proposed framework on handle, sculpture, and cat‐shaped objects, as shown in Figure 7. These samples contain more irregular contours and local structural features than the planar letter and toy targets, including elongated boundaries, head and shoulder profiles, pointed ears, and recessed regions. The DH channel again produces spatially separated or stretched replicas that encode axial information but degrade the interpretability of object texture. The Airy channel, by contrast, provides a more compact observation of the object outline and serves as the basis for subsequent texture recovery.

**FIGURE 7:**
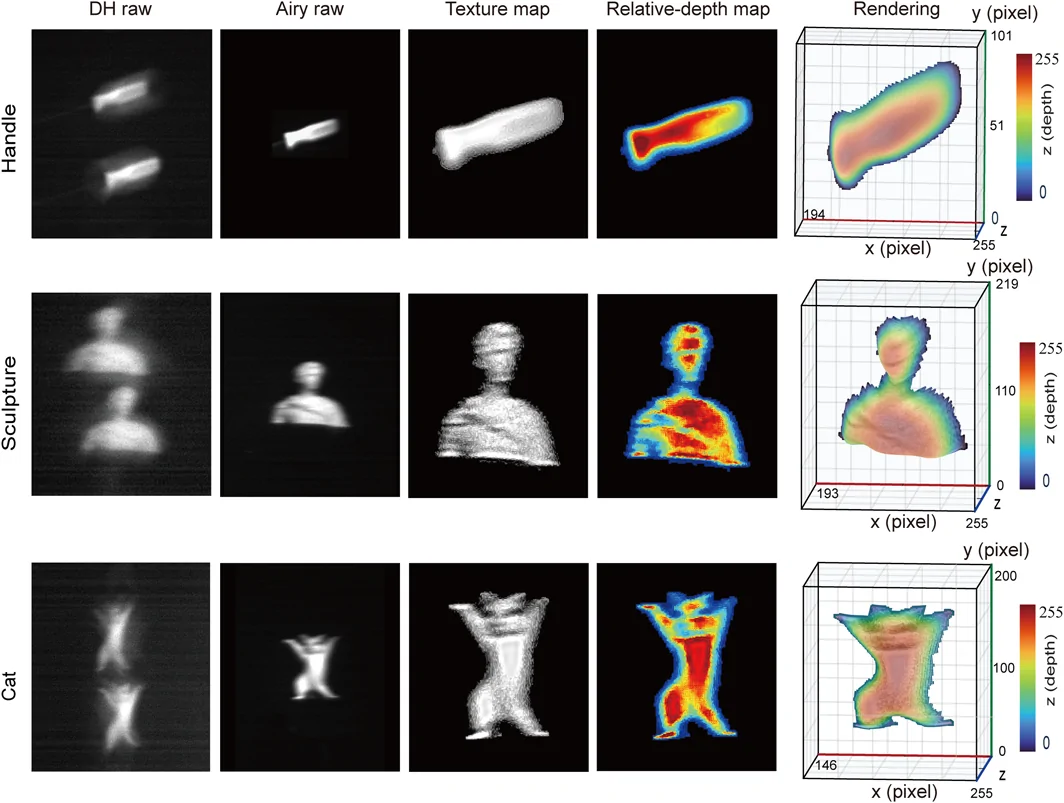
Depth–texture reconstruction of complex objects. Rows show the handle, sculpture, and cat‐shaped objects. Columns show the raw DH and Airy observations, recovered texture maps, relative‐depth maps, and the resulting three‐dimensional rendering.

For these irregular objects, the Airy images were processed by background suppression and PSF‐constrained non‐blind deconvolution to recover texture maps within the object support. A small fraction of the original Airy intensity was retained during restoration to preserve weak texture components and edge details. Relative‐depth maps were then constructed within the Airy‐defined object contours and combined with the recovered textures to generate the final depth–texture representations. The reconstructed results preserve the global object outlines as well as local appearance features that are strongly degraded in the raw DH observations.

These experiments extend the validation from controlled multi‐depth targets to more complex object geometries. The resulting relative‐depth maps describe local variations within the Airy‐defined object support, whereas the recovered Airy textures preserve the corresponding object contours and appearance features. The final column presents depth‐guided three‐dimensional renderings in which the relative surface relief is displayed with the corresponding recovered texture.

## 3 Conclusion

We report a polarization‐multiplexed metalens that encodes a depth‐sensitive double‐helix PSF and a compact Airy PSF into the left‐ and right‐handed circular polarization channels, respectively. This architecture decouples depth encoding from texture observation within a single TiO_2_ planar device, thereby avoiding the need for one spatially extended PSF to perform both functions. The fabricated metalens has a 1 mm aperture, a 10 mm focal length, and a design wavelength of 532 nm. Experimental characterization showed that the Airy channel provided an approximately 11‐fold extension of the depth of field relative to conventional focused imaging, whereas the DH feature enabled calibrated depth estimation over a tested working distance of 20–70 cm, with a minimum tested axial separation of 2 cm. In cases where the two DH replicas strongly overlapped, the Airy observation also served as an internal structural template for extracting the encoded peak spacing. The recovered depth and Airy‐derived texture were subsequently combined to reconstruct multi‐depth patterns and complex objects with improved boundary and appearance preservation. More broadly, these results show that the depth–texture trade‐off in PSF‐engineered imaging can be mitigated through channel‐function decoupling. The proposed depth–texture co‐design strategy may be extended to other complementary PSFs and integrated with broadband meta‐optics and computational reconstruction methods, providing a compact route toward passive depth‐resolved and texture‐preserving three‐dimensional imaging.

## 4 Methods

### 4.1 Metalens Fabrication

The numerical simulation of the metalens was performed using the finite‐difference time‐domain (FDTD) method. The fabrication process was as follows: a 600 nm‐thick TiO_2_ film was deposited on a fused‐silica substrate, with a nanopillar pitch of 400 nm and an aperture diameter of 1 mm. Approximately 200 nm‐thick ZEP‐520A electron‐beam resist was spin‐coated and patterned by electron‐beam lithography and development, followed by deposition of an approximately 50 nm Cr layer as a hard mask. The resist was then removed by lift‐off in N‐methyl‐2‐pyrrolidone (NMP) solution to expose the Cr mask pattern. The pattern was transferred into the TiO_2_ layer by inductively coupled plasma reactive‐ion etching (ICP‐RIE), and the Cr hard mask was finally removed using a Cr etchant. After fabrication, scanning electron microscopy was used to examine the geometry and arrangement quality of the nanopillars.

### 4.2 Optical Acquisition System

For the DH and Airy PSF characterization in Figure 4, the LED emission was collected and focused by an achromatic doublet with a focal length of 30 mm and a microscope objective onto a 15‐μm‐diameter pinhole. The pinhole therefore served as the effective spatial point source and suppressed the spatial structure of the LED emitter. A wavefront‐conditioning lens was placed between the pinhole and the metalens. During the axial PSF measurements, the lens‐to‐metalens distance was kept fixed, while the point‐source module was translated axially to vary the spherical‐wave curvature incident on the metalens and emulate different object depths. The resulting PSFs were collected by an objective and relayed through an achromatic tube lens and a spectral filter to the complementary metal–oxide–semiconductor (CMOS) camera.

The experimental optical path used for target imaging is shown in Figure 5a. The illumination arm consisted of an LED source, a linear polarizer (LP) and a quarter‐wave plate (QWP), which generated circularly polarized illumination on the measured target. The target field was relayed by TL1 and then projected by Obj.1 to form a reduced‐size intermediate image at the object‐side working plane of the metalens. After modulation by the metalens, Obj.2 collected the output field, while QWP2 and LP2 selected the desired polarization channel. TL2 then relayed the image through a 532‐nm narrow‐band filter onto a CMOS camera (MER2‐230‐168U3C) with a pixel size of 5.86 μm × 5.86 μm. In the calibration experiment, a checkerboard target was placed at known depths using a precision translation stage, and paired images were acquired to establish the depth‐calibration curve.

### 4.3 Airy‐Template‐Constrained Point‐Cloud Reconstruction and Texture Refinement

After depth inversion, the raw Airy image was deconvolved using the Airy PSF selected at the DH‐estimated axial position to recover the texture $T_{A}\left(x,y\right)$. The object support $M_{A}\left(x,y\right)$ was then obtained by adaptive thresholding, connected‐component selection, and boundary cleaning. For object m, the point cloud was constructed as

(6) $$P_{m}=\left\{\left(\left({\left[X_{m}\left(x,y\right),Y_{m}\left(x,y\right),{\hat{z}}_{m}\right]}^{T},T_{A}^{\left(m\right)}\left(x,y\right)\right)|M_{A}^{\left(m\right)}\left(x,y\right)=1\right)\right\},$$

where (*x*, *y*) are image‐plane pixel coordinates; $X_{m}\left(x,y\right)$ and $Y_{m}\left(x,y\right)$ are the lateral coordinates; ${\hat{z}}_{m}$ is the axial position inferred from the DH twin‐peak spacing; $T_{A}^{\left(m\right)}\left(x,y\right)$ is the recovered Airy texture; and $M_{A}^{\left(m\right)}\left(x,y\right)$ is the valid Airy support. Retaining only pixels within this support suppresses the expanded boundary of the DH replicas and constrains the point‐cloud outline to the Airy observation.

For the enhanced appearance branch used with complex textured objects, an aligned reference appearance was introduced only as a confidence‐gated residual correction to the measured Airy texture:

(7) $$T_{r}=\Pi_{\left[0,1\right]}\left[T_{A}+A_{g}⊙\Delta T_{\text{ref}}\right],$$

here $T_{r}$ is the refined texture, $\Pi_{\left[0,1\right]}$ clips the normalized intensity range, the symbol ⊙ denotes element‐wise multiplication, and $A_{g}$ in [0, 1] is a soft confidence map derived from the overlap between the Airy support and the reference aligned through aspect‐ratio‐preserving scaling and centroid translation. The residual correction was decomposed into low‐ and high‐spatial‐scale components:

(8) $$\Delta T_{\text{ref}}=\lambda_{1}\left(G_{\text{low}}-T_{A,\text{low}}\right)+\lambda_{h}G_{\text{high}}.$$

$T_{A,\text{low}}$ denotes the smoothed Airy texture, $G_{\text{low}}$ and $G_{\text{high}}$ are the low‐ and high‐spatial‐scale components of the aligned reference, and $\lambda_{1}$ and $\lambda_{h}$ control their contributions. The correction is restricted to confidence‐supported regions.

The final rendering color is jointly determined by the depth‐encoded color and the recovered texture appearance:

(9) $$C_{k}=\Pi_{\left[0,1\right]}\left\{\left[\left(1-\alpha \right)C_{Z}\left(z_{k}\right)+\alpha C_{T}\left(t_{k}\right)\right]L\left(t_{k}\right)\right\},$$

where $C_{Z}\left(z_{k}\right)$ denotes the depth‐colormap value assigned to the kth point, $C_{T}\left(t_{k}\right)$ represents the grayscale appearance derived from its texture intensity, and α is the mixing coefficient between the two components. $L\left(t_{k}\right)$ modulates the local brightness and contrast.

## Author Contributions

**Xinyi Tian:** conceptualization, methodology, validation, formal analysis, writing – original draft. **Zhanhong Zhou:** writing – review and editing, formal analysis. **Bohan Xiao:** visualization, writing – review and editing. **Li Yao:** writing – review and editing. **Zhihong Liu:** visualization. **Tianyun Wang:** formal analysis, validation. **Yuhong Luo:** visualization. **Jun Wang:** visualization. **Yujie Xia:** visualization. **Chao Shen:** resources. **Junwen Zhang:** resources. **Nan Chi:** resources. **Ziwei Li:** conceptualization, funding acquisition, writing – review and editing, supervision.

## Supporting information


Supporting Information S1

## Data Availability

The data that support the findings of this study are available on request from the corresponding author. The data are not publicly available due to privacy or ethical restrictions.
