# Supplementary material for: Polarization‐Multiplexed Double‐Helix/Airy Metalens for Depth‐Resolved Texture Imaging With Extended Depth‐of‐Field
Source: Nanophotonics. 2026 Oct 6;15(19):e70311. doi: 10.1002/nap2.70311 (PMC13643062; doi:10.1002/nap2.70311)
Supplement: Supplementary file 1 [file NAP2-15-e70311-s001.docx]

Supporting Information

**Polarization-Multiplexed Double-Helix/Airy Metalens for Depth-**

resolved Texture Imaging with Extended Depth-of-Field

*Xinyi Tian, Zhanhong Zhou, Bohan Xiao, Li Yao, Zhihong Liu, Tianyun Wang, Yuhong Luo, Jun Wang, Yujie Xia, Chao Shen, Junwen Zhang, Nan Chi, Ziwei Li*^*^

*Xinyi Tian, Zhanhong Zhou, Bohan Xiao, Li Yao, Zhihong Liu, Tianyun Wang, Yuhong Luo, Jun Wang, Yujie Xia, Chao Shen, Junwen Zhang, Nan Chi, Ziwei Li*

College of Future Information Technology, Fudan University

Shanghai 200433, China

E-mail: [lizw@fudan.edu.cn](mailto:lizw@fudan.edu.cn)

*Chao Shen, Junwen Zhang, Nan Chi, Ziwei Li*

Key Laboratory for Information Science of Electromagnetic Waves (MoE), Fudan University

Shanghai 200433, China

**Supporting Note 1.** **Phase response and transmittance of TiO_2_ meta-atoms**

Full-wave electromagnetic simulations were performed to construct the meta-atom library used for the polarization-multiplexed metalens. As shown in Supplementary Figure. S1(a), each unit cell consists of a rectangular TiO_2_ nanopillar on a SiO_2_ substrate. The nanopillar length *L* and width *W* define the in-plane anisotropy of the meta-atom, while the lattice period *P* and height *H* are fixed according to the device design. The optical constants of TiO_2_ and SiO_2_ used in the simulations were taken from the material dispersion data, and the wavelength-dependent refractive index *n* and extinction coefficient *k* of TiO_2_ are shown in Supplementary Figure. S1(b).

To evaluate the polarization-dependent optical response, the geometric parameters $L$and $W$ were swept over the designed parameter space. Periodic boundary conditions were applied along the transverse *x*- and *y*-directions, and perfectly matched layers were used along the propagation direction. Normally incident plane waves polarized along the *x*- and *y*-axes were used to calculate the transmitted phase delays and, together with the corresponding transmittances $T_{x}$ and $T_{y}$.

Supplementary Figure S1(c,d) shows the simulated phase shifts for *x*- and *y*-polarized incidence as functions of $L$ and $W$. The TiO_2_ nanopillars provide a broad and continuous phase modulation range, covering nearly the full $2\pi$ phase space for both orthogonal linear polarizations. Supplementary Figure S1(e,f) presents the corresponding transmission maps. Although resonant regions introduce rapid phase variations and reduced transmission, a large parameter region maintains high transmittance while still offering sufficient phase tunability.

The simulated phase and transmission maps were used as a lookup table for selecting meta-atoms. For each spatial position of the metalens, the nanopillar geometry was chosen to best match the target phase delays required by the two polarization channels while maintaining high transmission efficiency. This procedure enables independent wavefront control for the

orthogonal polarization components and provides the physical basis for implementing the double-helix and Airy point-spread-function responses within a single TiO_2_ planar device.

**Figure S1.** Simulated optical response of TiO_2_ meta-atoms. (a) TiO_2_ nanopillar unit cell on a SiO_2_ substrate. (b) Wavelength-dependent refractive index *n* and extinction coefficient *k* of TiO_2_. (c,d) Simulated phase shifts and, and (e,f) transmittances $T_{x}$ and $T_{y}$, as functions of nanopillar length $L$ and width $W$.

**Supporting Note 2. Design principle of metasurfaces**

The analytical phase functions used to construct the three constituent profiles are defined below. The common focusing phase is

$$\text{Φ}_{focus}(x,y)=k\left(\sqrt{f^{2}+x^{2}+y^{2}}-|f|\right)$$

The two opposite steering contributions are described by

$$\text{Φ}_{tilt}^{+}(x,y)=k\left[\sqrt{f^{2}+x^{2}+y^{2}+1.5xf\sin\theta_{s}}-\sqrt{f^{2}+x^{2}+y^{2}}\right]$$

$$\text{Φ}_{tilt}^{-}(x,y)=k\left[\sqrt{f^{2}+x^{2}+y^{2}-1.5xf\sin\theta_{s}}-\sqrt{f^{2}+x^{2}+y^{2}}\right]$$

Here, *x* and *y* denote the transverse aperture coordinates, $f$ is the design focal length, and $\theta_{s}$ is the steering angle. The positive and negative linear-gradient phases are

$$\text{Φ}_{\text{G}}^{\pm}(x,y)=\pm\frac{2\pi}{\lambda}x\sin\alpha$$

where k = -2π/λ is the free-space wave number and α = 7° is the designed gradient angle. The complementary Fresnel-zone azimuthal phase is written as:

where$r=\sqrt{x^{2}+y^{2}}$ and $\theta=\arctan(y/x)$ are the polar coordinates, $R_{\max}$is the aperture radius, $L$ is the maximum azimuthal order, and $\varepsilon$ controls its radial variation. The two complementary branches are obtained using $q=0$ for $\Phi_{F}^{0}$ and $q=1$ for $\Phi_{F}^{1}$. Outside the clear aperture, the phase is set to zero. The cubic phase used in the Airy channel is:

$$\text{Φ}_{cubic}(x,y)=\pi\left[\left(\frac{x}{R_{\max}}\right)^{3}+\left(\frac{y}{R_{\max}}\right)^{3}\right]$$

For the designed metasurface, the period of the TiO₂ nanopillars is set to *P* = 400 nm. With this subwavelength period, each nanofin operates as a zero-order grating, which suppresses high-order diffraction and maintains high transmittance. Polarization multiplexing requires each nanofin to provide independent phase modulation along its two principal axes under linearly polarized illumination, giving rise to a shape-dependent birefringence effect. The nanopillar length *L* and width *W* are swept from 100 nm to 250 nm with a 5 nm step for each geometric parameter. Supplementary Figure S1(a,b) presents the co-polarized transmittance and phase response of the nanopillars. The dependence of the transmittance and phase response on *L* and *W* for *x*- and *y*-polarized incidence is calculated using the finite-difference time-domain (FDTD) method.

For circular polarization multiplexing, the target phase profiles defined in the main text,$\Phi_{LCP}(x,y)$and $\Phi_{RCP}(x,y)$, are encoded in two orthogonal spin channels of the same metasurface. We use the fixed circular states $|k_{+}\rangle=\left(1,i\right)^{T}/\sqrt{2}$and $|k_{-}\rangle=\left(1,-i\right)^{T}/\sqrt{2}$. The incident $|k_{+}\rangle$ channel is assigned $\Phi_{LCP}(x,y)$, whereas the incident $|k_{-}\rangle$ channel is assigned $\Phi_{RCP}(x,y)$. An ideal half-wave anisotropic element converts each incident spin state into the orthogonal output spin state while imparting the corresponding target phase. Therefore, the Jones matrix $J(x,y)$ of the spin-multiplexing metasurface must satisfy the following conditions:

$$\left\{\begin{matrix} J(x,y)|k_{+}\rangle=e^{i\Phi_{LCP}(x,y)}|k_{-}\rangle\\ J(x,y)|k_{-}\rangle=e^{i\Phi_{RCP}(x,y)}|k_{+}\rangle\end{matrix}\right.$$

For compactness, let $a=e^{i\Phi_{LCP}(x,y)}$ , $b=e^{i\Phi_{RCP}(x,y)}$.

Accordingly, $J(x,y)$ can be expressed as :

$$J(x,y)=\frac{1}{2}\left[\begin{matrix} a+b & i(b-a) \\ i(b-a) & -(a+b) \end{matrix}\right]$$

The eigenvalues are obtained from the characteristic equation of $J(x,y)$ as:

$$\begin{matrix} \xi_{1}=e^{i\left[\frac{1}{2}\left(\Phi_{LCP}(x,y)+\Phi_{RCP}(x,y)\right)\right]} \\ \xi_{2}=e^{i\left[\frac{1}{2}\left(\Phi_{LCP}(x,y)+\Phi_{RCP}(x,y)\right)-\pi\right]} \end{matrix}$$

In addition, the eigenvectors are:

$$\begin{matrix} |\lambda_{1}\rangle=\left[\begin{matrix} \cos\frac{1}{4}\left[\Phi_{LCP}(x,y)-\Phi_{RCP}(x,y)\right] \\ \sin\frac{1}{4}\left[\Phi_{LCP}(x,y)-\Phi_{RCP}(x,y)\right] \end{matrix}\right] \\ |\lambda_{2}\rangle=\left[\begin{matrix} -\sin\frac{1}{4}\left[\Phi_{LCP}(x,y)-\Phi_{RCP}(x,y)\right] \\ \cos\frac{1}{4}\left[\Phi_{LCP}(x,y)-\Phi_{RCP}(x,y)\right] \end{matrix}\right] \end{matrix}$$

Thus, under the symmetric and unitary conditions, $J(x,y)$ can be written in the standard form:

$$J(x,y)=P\Lambda P^{-1}=\left[\begin{matrix} \lambda_{1} & \lambda_{2} \end{matrix}\right]\left[\begin{matrix} \xi_{1} & 0 \\ 0 & \xi_{2} \end{matrix}\right]\left[\begin{matrix} \lambda_{1} & \lambda_{2} \end{matrix}\right]^{-1}$$

In this context, $P$ is a real-invertible matrix, while Λ is a diagonal matrix. Each unit cell of the metasurface can be considered as a birefringent optical element, with the diagonal matrix Λ governing the phase delays and along the two perpendicular symmetry axes, corresponding to the two orthogonal principal axes of the nanofin. The matrix $P$ defines the rotation angle *θ* of these fast axes. Specifically, and describe the propagation phase delays. Given the spin-multiplexed phase profiles, the required phase shifts and rotation angles can be derived.

**Supporting Note 3. Simulated comparison between DH + ordinary lens and DH + Airy lens**

To verify the advantage of using an Airy phase as the texture-imaging channel, two multiplexed phase designs were simulated under the same aperture, NA, and propagation distance. In the first design, the DH phase was combined with an ordinary focusing lens. In the second design, the same DH phase was combined with an Airy-lens phase.

As shown in Supplementary Figure S2, both designs preserve the DH response for depth encoding. Compared with the ordinary focusing channel, the Airy-modulated channel exhibits lower sensitivity to axial defocus and preserves a more consistent target morphology over the investigated object-distance range. The reduced distance-dependent image degradation verifies the extended-depth-of-field capability introduced by the Airy phase. Consequently, the Airy response serves as a robust complementary channel for texture observation across varying object distances, while the DH response provides depth-sensitive encoding.

**Figure S2.** Simulated object-distance response of the DH/ordinary-lens and DH/Airy-lens designs.

**Supporting Note 4. Near-distance imaging setup for spin-channel validation**

To verify the basic imaging capability and spin-dependent response of the metalens, a near-distance imaging setup was constructed, as shown in Supplementary Figure S3(a). The illumination polarization was controlled by a linear polarizer and a quarter-wave plate before the target, and the output polarization was analyzed after the metalens. The image was relayed to the CMOS camera by an objective and a tube lens.

Supplementary Figure S3(b) shows the captured images of an “F” target under different incident polarization states. Under linearly polarized illumination, both circular-polarization channels are excited, leading to a superposed imaging response. Under RCP and LCP illumination, different image patterns are selectively obtained, confirming the spin-dependent optical response of the metalens. This result verifies that the two polarization channels can be independently addressed, which provides the experimental basis for assigning different PSF functions to the two spin states.

Supplementary Figure S3(c) illustrates the corresponding imaging geometry, where the object distance $a$, image-side distance $b$, and focal length $f$ of a single metalens follow the Gaussian lens formula.

**Figure S3.** Near-distance spin-dependent imaging. (a) Experimental setup. (b) “F” target images under LP, RCP, and LCP illumination. (c) Imaging geometry, where $a$ and $b$ denote the object-side and image-side distances of the metalens.

**Supporting Note 5. Simulated efficiencies of the DH and Airy channels**

In the simulation, a linearly polarized input was used so that the two circular-polarization components were excited simultaneously with equal incident power. After polarization-resolved decomposition, the DH and Airy components were propagated to the sensor plane, and equal-sized spatial regions of interest (ROIs) were used for the two channels. The simulated channel efficiency was defined as $\eta_{j}=\frac{P_{j,ROI}}{P_{j}}$.

Where, $P_{j,ROI}$ is the optical power contained within the target-PSF ROI and $P_{j}$ is the total focal-plane power of the corresponding polarization channel. Using the same ROI size for both channels, the simulated channel efficiencies are 75.11% for the DH channel and 99.57% for the Airy channel. The lower DH-channel efficiency is mainly associated with its more spatially extended double-lobe response and the presence of off-target diffracted components and sidelobe energy, which cause a fraction of the channel power to fall outside the selected ROI. In contrast, the Airy-channel energy is more strongly concentrated within the target region.

**Figure S4**. Simulated efficiencies of the DH and Airy channels. (a) Total PSF under linearly polarized illumination. (b,c) DH and Airy PSFs within equal-sized ROIs. (d) Simulated channel efficiencies.

**Supporting Note 6. PSF size and MTF comparison with an ordinary focusing lens**

The Airy and ordinary focusing channels were simulated using the same wavelength, focal length and effective aperture. At the nominal focal plane, the Airy main-lobe FWHMs are 47.3 and 47.2 μm along x and y, respectively, compared with 50.8 μm in both directions for the ordinary focusing channel. The corresponding best-focus MTF50 is approximately 5.6-5.9 lp/mm for the Airy channel and 8.2 lp/mm for the ordinary channel. The ordinary-channel MTF50 decreases more rapidly with defocus, whereas the Airy-channel response varies more slowly. The EDOF extension therefore involves a moderate reduction in the best-focus spatial-frequency response in exchange for improved defocus tolerance, rather than a pronounced increase in the main-lobe size.

**Figure S5**. Simulated comparison of the Airy and conventional focusing channels under identical optical conditions. (a,b) Normalized PSFs of the Airy and conventional focusing channels at the nominal focal plane. (c) Corresponding transverse PSF profiles. (d) MTF comparison at the nominal focal plane. (e,f) MTF50 as a function of defocus in the x and y directions, respectively.

**Supporting Note 7. Depth-estimation error metrics**

For each target, the relative depth error was calculated as

$$\varepsilon_{i}=\frac{\left|z_{i}^{est}-z_{i}^{GT}\right|}{z_{i}^{GT}}\times100\text{\%},$$

Where $z_{i}^{est}$ and $z_{i}^{GT}$denote the estimated and ground-truth depths of the $i$-th target, respectively. The normalized mean absolute error (NMAE) over $N$targets was defined as

$$NMAE=\frac{1}{N}\sum_{i=1}^{N} \frac{\left|z_{i}^{est}-z_{i}^{GT}\right|}{z_{i}^{GT}}\times100\text{\%}.$$

**References**

[1]	Z. Shen, F. Zhao, C. Jin, S. Wang, L. Cao, and Y. Yang, “Monocular metasurface camera for passive single-shot 4D imaging,” *Nature Communications* 14 no. 1 (2023): 1035, https://doi.org/10.1038/s41467-023-36812-6.

[2]	G. A. Siviloglou, and D. N. Christodoulides, “Accelerating finite energy Airy beams,” *Optics Letters* 32 no. 8 (2007): 979–981, https://doi.org/10.1364/ol.32.000979.

[3]	E. R. Dowski, and W. T. Cathey, “Extended depth of field through wave-front coding,” *Applied Optics* 34 no. 11 (1995): 1859–1866, https://doi.org/10.1364/ao.34.001859.
